# Effects of low dose GM-CSF on microglial inflammatory profiles to diverse pathogen-associated molecular patterns (PAMPs)

**DOI:** 10.1186/1742-2094-4-10

**Published:** 2007-03-20

**Authors:** Nilufer Esen, Tammy Kielian

**Affiliations:** 1 Department of Neurobiology and Developmental Sciences, University of Arkansas for Medical Sciences, Little Rock, AR 72205, USA

## Abstract

**Background:**

It is well appreciated that obtaining sufficient numbers of primary microglia for *in vitro *experiments has always been a challenge for scientists studying the biological properties of these cells. Supplementing culture medium with granulocyte-macrophage colony-stimulating factor (GM-CSF) partially alleviates this problem by increasing microglial yield. However, GM-CSF has also been reported to transition microglia into a dendritic cell (DC)-like phenotype and consequently, affect their immune properties.

**Methods:**

Although the concentration of GM-CSF used in our protocol for mouse microglial expansion (0.5 ng/ml) is at least 10-fold less compared to doses reported to affect microglial maturation and function (≥ 5 ng/ml), in this study we compared the responses of microglia derived from mixed glial cultures propagated in the presence/absence of low dose GM-CSF to establish whether this growth factor significantly altered the immune properties of microglia to diverse bacterial stimuli. These stimuli included the gram-positive pathogen *Staphylococcus aureus *(*S. aureus*) and its cell wall product peptidoglycan (PGN), a Toll-like receptor 2 (TLR2) agonist; the TLR3 ligand polyinosine-polycytidylic acid (polyI:C), a synthetic mimic of viral double-stranded RNA; lipopolysaccharide (LPS) a TLR4 agonist; and the TLR9 ligand CpG oligonucleotide (CpG-ODN), a synthetic form of bacteria/viral DNA.

**Results:**

Interestingly, the relative numbers of microglia recovered from mixed glial cultures following the initial harvest were not influenced by GM-CSF. However, following the second and third collections of the same mixed cultures, the yield of microglia from GM-CSF-supplemented flasks was increased two-fold. Despite the ability of GM-CSF to expand microglial numbers, cells propagated in the presence/absence of GM-CSF demonstrated roughly equivalent responses following *S. aureus *and PGN stimulation. Specifically, the induction of tumor necrosis factor-α (TNF-α), macrophage inflammatory protein-2 (MIP-2/CXCL2), and major histocompatibility complex (MHC) class II, CD80, CD86 expression by microglia in response to *S. aureus *were similar regardless of whether cells had been exposed to GM-CSF during the mixed culture period. In addition, microglial phagocytosis of intact bacteria was unaffected by GM-CSF. In contrast, upon *S. aureus *stimulation, CD40 expression was induced more prominently in microglia expanded in GM-CSF. Analysis of microglial responses to additional pathogen-associate molecular patterns (PAMPs) revealed that low dose GM-CSF did not significantly alter TNF-α or MIP-2 production in response to the TLR3 and TLR4 agonists polyI:C or LPS, respectively; however, cells expanded in the presence of GM-CSF produced lower levels of both mediators following CpG-ODN stimulation.

**Conclusion:**

We demonstrate that low levels of GM-CSF are sufficient to expand microglial numbers without significantly affecting their immunological responses following activation of TLR2, TLR4 or TLR3 signaling. Therefore, low dose GM-CSF can be considered as a reliable method to achieve higher microglial yields without introducing dramatic activation artifacts.

## Background

Granulocyte-macrophage colony-stimulating factor (GM-CSF) is a well known hematopoietic cytokine produced primarily by T cells, macrophages, endothelial cells, and fibroblasts [1-4]. GM-CSF was originally defined based on its ability to stimulate the differentiation and function of granulocytes, monocytes and macrophages [1]. In addition, previous studies have established that GM-CSF promotes the survival and proliferation of neonatal rat, mouse, and human microglia in culture [5-9]. Based on these observations, GM-CSF is commonly used as a culture medium supplement to obtain sufficient numbers of microglia to conduct downstream *in vitro *experiments [10-12]. However, recent studies have suggested that microglia are not terminally differentiated and that GM-CSF can induce their functional maturation and expression of dendritic cell (DC) markers [13-15], which has raised concerns with investigators who are examining the immunological functions of primary microglia in various CNS pathologies. For example, GM-CSF has been reported to induce the transcription of genes important for T cell activation, chemotaxis, antigen processing, innate immunity, and immunosuppression, suggesting the transition of microglia into a more professional antigen presenting cell phenotype [14-16]. In addition, other studies have utilized GM-CSF to induce DC maturation from myeloid progenitor cells [17,18]. Overall, these studies suggest that microglia can transition into a DC-like phenotype when cultured in the presence of adequate levels of GM-CSF.

During the preparation of our mouse primary mixed glial cultures, we routinely supplement culture medium with low levels of GM-CSF (0.5 ng/ml) to increase microglial yields. Despite the fact that this concentration is approximately ten-fold lower than what has been reported to modulate microglial function and transition into a DC phenotype (i.e. 5–50 ng/ml), we are often questioned regarding the consequences of microglial exposure to GM-CSF during the mixed glial culture period and whether this introduces artifacts in the activation profiles of these cells in subsequent *in vitro *experiments. Therefore, the primary objective of the present study was to evaluate whether low dose GM-CSF leads to alterations in microglial morphology and/or functional activation in response to a wide variety of PAMPs commonly associated with various CNS infections, namely *Staphylococcus aureus *(*S. aureus*) and its cell wall component peptidoglycan (PGN), LPS, polyI:C, and CpG-ODN. Our results demonstrate that low dose GM-CSF led to a significant expansion in microglial numbers without affecting their phagocytic activity or cytokine production profiles in response to the majority of PAMPs examined, with the exception of the TLR9 agonist CpG-ODN. However, we did observe a phenotypic transformation of microglia expanded in the presence of low dose GM-CSF, namely a transition to a DC-like morphology typified by numerous dendrites; however, the functional implication(s) of this change remain to be determined. Therefore, low dose GM-CSF can be successfully utilized as a culture medium supplement to enhance microglial recovery without overtly compromising normal responsiveness to microbial stimuli. Importantly, these findings exclude any potential GM-CSF-induced artifacts in the read-outs of microglial activation routinely used in our studies.

## Methods

### Primary microglia cell culture and reagents

Primary microglia were isolated from inbred neonatal C57BL/6 mice as previously described [19]. Briefly, age-matched litters (postnatal day 2–5) were euthanized using an overdose of inhaled Halothane (Halocarbon Laboratories, River Edge, NJ) to obtain mixed glial cultures. Cerebri were collected under aseptic conditions and the meninges removed. Tissues were minced, resuspended in trypsin-EDTA (Mediatech Inc., Herndon, VA), and incubated at 37°C for 20 minutes. Subsequently, cells were resuspended in complete DMEM (4.5 g/L glucose, Mediatech Inc.) containing 10% FBS (Hyclone, Logan, UT), 200 mM L-glutamine, 100 U/ml penicillin, 0.1 mg/ml streptomycin and 0.25 μg/ml fungizone (all from Mediatech Inc.), OPI supplement (oxalacetic acid, pyruvate, insulin; Sigma, St. Louis, MO), and 0.5 ng/ml recombinant mouse GM-CSF (BD Pharmingen, San Diego, CA). The cell suspension was further triturated and filtered through a 70 μm cell strainer. Subsequently, cells were centrifuged, resuspended in complete medium, and seeded into 150 cm^2 ^flasks. To minimize variation between microglia expanded in the presence/absence of GM-CSF, mouse pups were procured from litters born on the same day and primary cultures propagated with or without GM-CSF were derived from the same initial mixed glial population (i.e. half of the mixed glial cells recovered were cultured with GM-CSF (+) medium while the other half was cultured without GM-CSF).

Upon confluence (7–10 days), flasks were shaken overnight at 200 rpm at 37°C to recover microglia. Microglia from both (+) GM-CSF- and (-) GM-CSF-treated flasks were collected and plated in medium without GM-CSF for all subsequent experiments. The purity of microglial cultures was evaluated by immunohistochemical staining using antibodies against CD11b and GFAP to identify microglia and astrocytes, respectively, and was routinely greater than 95%. Each experiment presented in this paper was initially performed with microglia collected after the first shake and repeated with cells collected after a second shake to confirm that the responses were comparable. Based on our findings that microglial responses were similarly affected in all experiments regardless of when they were harvested, we concluded that microglial responsiveness to microbial stimuli does not significantly differ in cells collected from the first versus subsequent shakes.

Heat-inactivated *S. aureus *(strain RN6390) was prepared as previously described [11] and PGN derived from *S. aureus*, poly I:C, and the synthetic CpG oligonucleotide ODN1826 were obtained from InvivoGen (San Diego, CA). *Escherichia coli *O11:B1 LPS was purchased from List Biological Laboratories (Campbell, CA). The doses of stimuli used throughout this report were based on our previous studies that established optimal cytokine responses induced following bacterial stimulation without any evidence of toxicity [20,21]. All non-LPS reagents were verified to have endotoxin levels < 0.03 EU/ml as determined by Limulus amebocyte lysate assay (Associates of Cape Cod, Falmouth, MA).

### Enzyme linked immunosorbent assay (ELISA)

Protein levels of TNF-α, IL-12p40 (OptEIA, BD Pharmingen) and macrophage inflammatory protein (MIP-2/CXCL2, DuoSet, R&D Systems, Minneapolis, MN) were quantified in conditioned medium from PAMP-stimulated microglia using ELISA kits according to the manufacturer's instructions (level of sensitivity = 15.6 pg/ml).

### Cell viability assays

To evaluate whether microglia expanded in the presence or absence of GM-CSF demonstrated similar survival profiles following bacterial activation, a standard MTT assay based upon the mitochondrial conversion of (3- [4,5-dimethylthiazol-2-yl]-2,5-diphenyl-tetrazolium bromide (MTT) into formazan crystals was performed as previously described [22].

### Phagocytosis assay

Primary microglia expanded with or without GM-CSF were seeded onto 12 mm coverslips in 24-well plates and incubated overnight. The following day, cells were treated with a heat-killed *S. aureus *isolate that constitutively expresses green fluorescence protein (GFP, kindly provided by Dr. Ambrose Cheung, Dartmouth Medical School) for 3 h, whereupon Hoechst 33342 (Molecular Probes, Eugene, OR) was added to visualize nuclei. Cells were washed extensively with PBS and incubated with 0.05% crystal violet in 0.15 M NaCl for 45 seconds to quench any fluorescence emitted by residual extracellular bacteria. Coverslips were viewed under fluorescence microscopy using an excitation wavelength of 460 – 490 nm (FITC filter, Olympus BX41, Tokyo, Japan).

### Immunofluorescence staining and confocal microscopy

Primary microglia expanded in the presence/absence of GM-CSF were seeded onto 12 mm coverslips in 24-well plates and incubated overnight. The following day, microglia were treated with 10^7 ^heat-inactivated *S. aureus *for 24 h, whereupon cells were washed extensively with PBS, fixed in ice-cold methanol, and incubated with PBS/10% donkey serum to prevent non-specific antibody binding (Jackson ImmunoResearch, West Grove, PA) for 30 min at room temperature. Subsequently, microglia were incubated with a MHC class-II antibody (rat anti-mouse, BD Pharmingen) overnight at 4°C. The following day, cells were incubated with a donkey anti-rat biotinylated secondary antibody (Vector Laboratories, Burlingame, CA) and detected using a streptavidin-Alexa Fluor 568 conjugate (Molecular Probes). Subsequently, a directly conjugated CD11b-FITC antibody (BD Pharmingen) was added and cells incubated for 1 h at 37°C, whereupon Hoechst 33342 was used to visualize nuclei. Controls included microglia incubated with secondary antibodies only to assess the extent of non-specific staining. Coverslips were imaged using a Zeiss laser scanning confocal microscope (LSM 510, Carl Zeiss Microimaging). Hoechst 33342 for nuclear visualization was excited by a 405 nm diode laser, FITC to visualize CD11b immunoreactivity was excited with a 488 nm argon laser, and Alexa Fluor 568 to demonstrate MHC class II expression was excited with a 561 nm DPSS laser, with images collected using the appropriate emissions. The confocal pinhole was set to obtain an optical section thickness of 1.6 μm. To demonstrate co-localization of CD11b, MHC class II, and Hoechst 33342 signals, RGB merges of individual confocal images were performed using the ImageJ software program (NIH Image).

### Flow cytometry

Primary microglia expanded in the presence/absence of GM-CSF were seeded into 6-well plates (2 × 10^6 ^cells/well), and incubated overnight. The following day, cells were treated with 10^7 ^heat-inactivated *S. aureus *for 24 h. At the end of the incubation period, cells were washed twice with PBS, and collected using a cell scraper. A total of 5 × 10^5 ^microglia in each group were stained for two-color flow cytometry using CD11b-FITC and CD11c-PE-Cy7 antibodies (both from BD Pharmingen). Fc receptors were blocked with the addition of Fc block™ (anti-CD16 and -CD32 cocktail, BD Pharmingen). In addition, a separate set of microglia were stained with antibodies directed against the co-stimulatory molecules CD40, CD80, and CD86, in addition to MHC class II (all from BD Pharmingen), and subsequently incubated with a donkey anti-rat IgG FITC-conjugated secondary antibody (Jackson ImmunoResearch Laboratories, West Grove, PA). Controls included microglia incubated with appropriate isotype control-matched antibodies to assess the extent of non-specific background staining. Cells were analyzed using a FACS Calibur cytometer (BD Biosciences, San Jose, CA) with settings based on the staining of microglia with isotype control antibodies alone.

### Morphological analysis

Primary microglia propagated in the presence/absence of GM-CSF were seeded into 35 mm dishes (2 × 10^6 ^cells/well), and incubated overnight. The following day, cells were treated with 10^7 ^heat-inactivated *S. aureus *for 24 h or left unstimulated. At the end of the incubation period, cell morphology was visualized by phase-contrast microscopy and images collected using a fixed stage upright epifluorescence microscope (BX51WI, Olympus) equipped with a 40 × water immersion objective lens and a 12-bit intensified monochrome CCD camera (CoolSnap ES, Photometrics, Tucson, AZ).

### Statistics

Significant differences between experimental groups were determined by the *t*-test for unequal variances at the 95% confidence interval using Sigma Stat (SPSS Science, Chicago, IL).

## Results

### Microglial recovery is enhanced following low dose GM-CSF treatment

It has been well established that GM-CSF exerts mitogenic effects on primary microglia, effectively expanding cell numbers [5,6,10,12,23]. Therefore, the use of this cytokine during the mixed glial culture period reduces the number of neonatal mice required to obtain sufficient numbers of microglia for subsequent studies. We routinely supplement our culture medium with a relatively low dose of GM-CSF; however, to date, we have not yet performed a detailed analysis regarding the efficacy of this dose on microglial expansion or more importantly, whether GM-CSF alters the subsequent responsiveness of microglia compared to cells that have never been exposed to exogenous growth factor. This became an essential issue to address since we are often questioned about the consequences of GM-CSF treatment on downstream microglial responses, thus forming the impetus for the current study. Work by others had demonstrated that GM-CSF led to the transition of microglia into macrophage and/or dendritic-like cells [13-15,18]; however, the doses of cytokine used to drive this differentiation (i.e. 10–50 ng/ml) ranged anywhere from 20- to 50-fold higher than the concentration used to expand microglia numbers in our experiments (i.e. 0.5 ng/ml). In addition, other important factors to consider include whether microglia are procured from neonatal versus adult animals or if microglia are expanded as mixed glial cultures or as purified cells.

**Figure 1:**
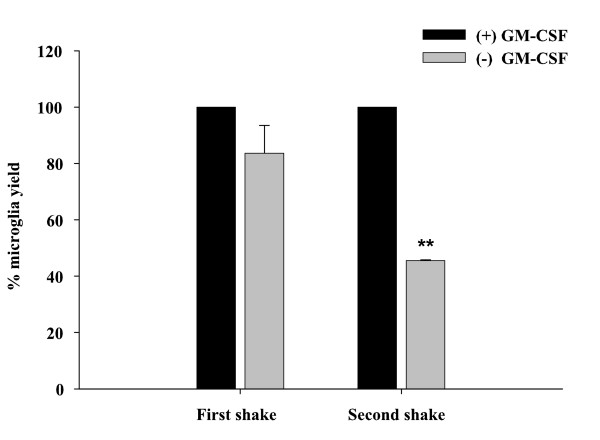
**GM-CSF improves microglial yields from primary cultures**. Mixed glial cells were prepared in culture medium supplemented with (+) or without (-) GM-CSF (0.5 ng/ml). Upon confluence (7–10 days), flasks were shaken overnight and the following day, supernatants were collected and microglial cell counts performed. Flasks were shaken once a week and following the second shake the relative percentage of microglia recovered from flasks cultured without GM-CSF was significantly reduced (**, *p *< 0.001). For reporting differences in microglial recovery, we normalized (i.e. divided) the numbers of microglia recovered from GM-CSF (-) flasks by those collected from GM-CSF (+) cultures to express the former as a percentage of GM-CSF (+)-containing conditions (set to 100%). Results represent the mean ± SD of two independent experiments.

To initiate our analysis of low dose GM-CSF effects on microglia, we quantified the relative percentages of microglia recovered from mixed glial cultures continuously propagated in the presence/absence of recombinant mouse GM-CSF (0.5 ng/ml). Upon reaching confluence, mixed glial cultures were collected at three consecutive harvests separated by an interval of 7–10 days. The number of microglia collected after the first harvest was not significantly influenced by GM-CSF; however, after the second and third harvests the number of microglia collected from GM-CSF-negative flasks was significantly lower compared to those supplemented with GM-CSF (Figure 1 and data not shown). This finding demonstrates that even at very low levels, GM-CSF is still capable of expanding microglial numbers, obviating the need for large numbers of neonatal animals to achieve sufficient cell yields.

### Low dose GM-CSF leads to morphological changes in microglia characteristic of dendritic cells (DCs)

Microglia display an ameboid morphology during embryogenesis [24,25] and assume a ramified shape upon maturation in the normal brain under physiological conditions [24,26,27]. However, in response to injury or infection, microglia become activated and transition into an ameboid morphology [27]. *In vitro*, ameboid microglia are rounded cells flattened to the substratum and have been reported to function as phagocytic antigen presenting cells [28, 29, 30, 31, 32]. This ameboid morphology observed *in vitro *is likely a consequence of the isolation procedure where, in general, ameboid characteristics are more typical of neonatal microglia, whereas adult cells normally exhibit a more quiescent ramified phenotype [33-36]. Nonetheless, ramification of ameboid microglia can be achieved by either growing cells on an astrocyte monolayer, culturing microglia in astrocyte-conditioned media, or treating with M-CSF [15,28,33].

**Figure 2:**
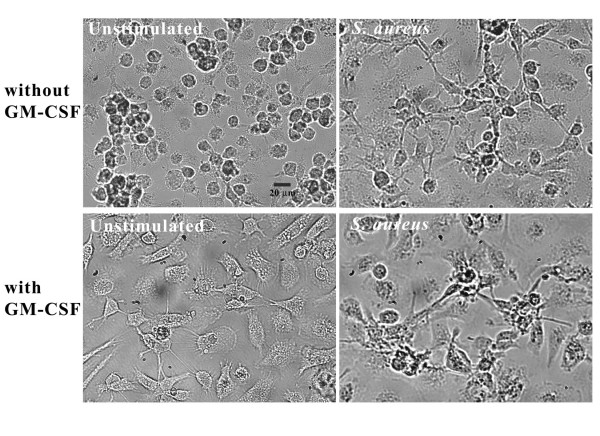
**Exposure of mixed glial cultures to low GM-CSF results in the ramification of resting microglia with a DC-like appearance. **Primary microglia expanded either with or without GM-CSF were seeded onto 35-mm dishes at 2 × 10^6 ^cells per dish and incubated overnight in 6-well plates. The following day, cells were either unstimulated or treated with heat-inactivated *S. aureus *(10^7 ^cfu/well) for 24 h, whereupon bright field phase-contrast images were collected (40×). The results pictured are representative of two independent experiments.

Although it is well established that GM-CSF induces microglial proliferation [5,6], there are conflicting reports in the literature regarding its effects on microglial morphology. For example, GM-CSF (5 U/ml for 72 h) has been shown to increase the number of ameboid microglia by 5- to 6-fold [6], whereas another study reported that GM-CSF induced microglial ramification and LPS treatment transformed cells to an ameboid phenotype [37]. To further complicate matters, recent studies have reported that exposure of purified adult microglia to GM-CSF led to their transformation into a macrophage- or DC-like morphology [13,15,38]. However, it is important to note that the relative concentrations of GM-CSF used in these studies were relatively high (i.e. 5–50 ng/ml) compared to our experiments (0.5 ng/ml). Therefore, to establish the effect of low dose GM-CSF on microglial morphology we investigated cells derived from our culture conditions by phase-contrast microscopy. As shown in Figure 2, unstimulated microglia expanded in the absence of GM-CSF appeared as single rounded or clustered cells that were relatively small in size. In contrast, microglia propagated in the presence of GM-CSF were more flattened, enlarged, and displayed a sizeable number of dendritic processes reminiscent of DCs (Figure 2) [15,38]. Interestingly, when stimulated with *S. aureus*, microglial morphology was virtually indistinguishable between cells that had been expanded in the presence/absence of GM-CSF (Figure 2). *S. aureus*-activated microglia appeared heterogeneous in shape compared to unstimulated cells typified by the presence of round, elongated, and flattened cells that exhibited the characteristic homotypic adhesion we have observed in our previous studies ([39] and unpublished observations). Collectively, these results indicate that exposure of mixed glial cultures to low dose GM-CSF leads to morphological alterations in purified "resting" microglia that are reminiscent of DCs, in agreement with studies by other groups using high concentrations of GM-CSF [15,33,38]. However, the morphological transformation associated with *S. aureus *activation of microglia is similar, regardless of prior exposure to GM-CSF.

### Microglial expansion with low dose GM-CSF leads to differential responses to various PAMPs

**Figure 3:**
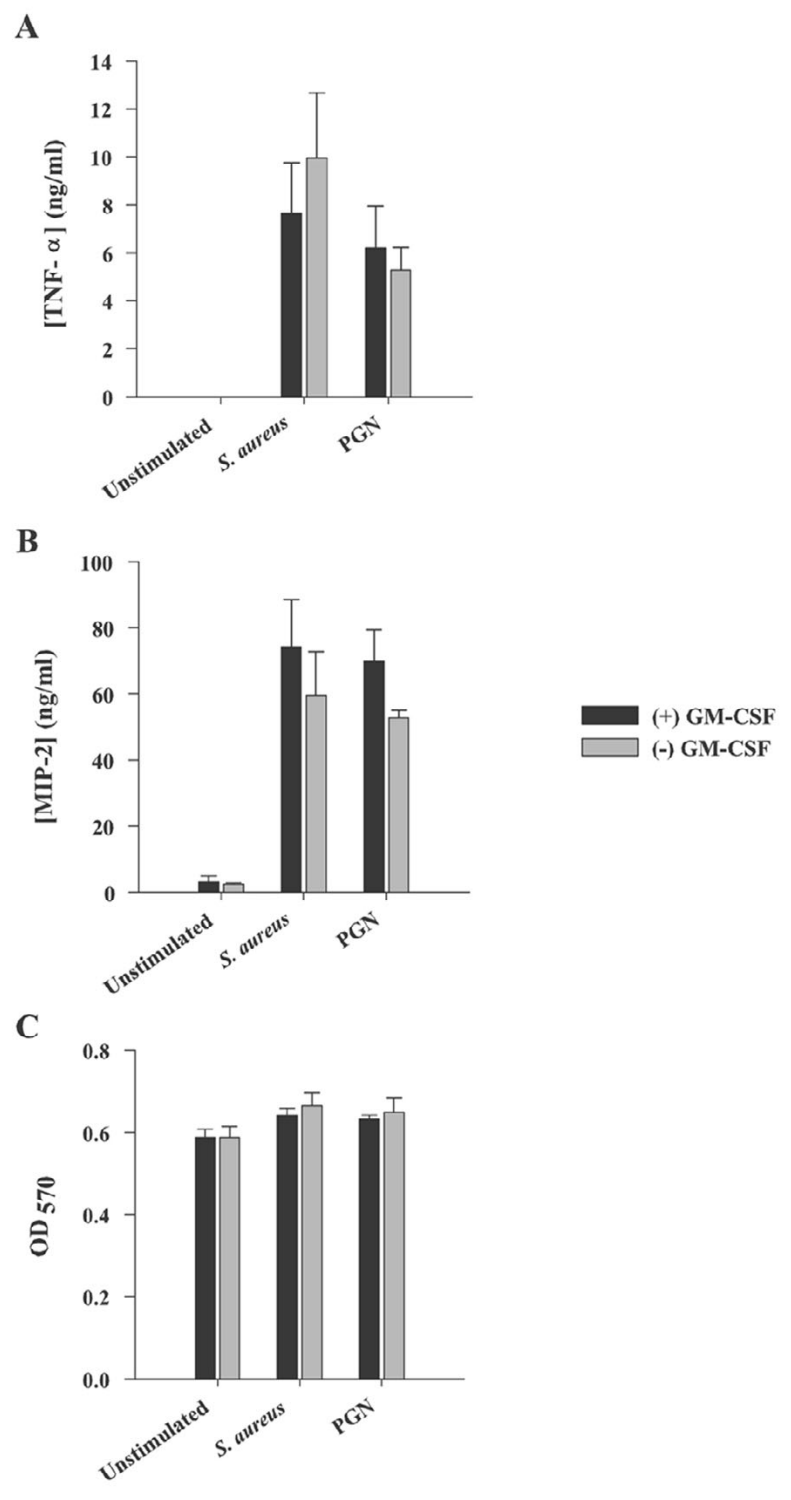
**Low dose GM-CSF does not alter microglial cytokine/chemokine responses to *S. aureus *and PGN**. Primary microglia expanded with (+) or without (-) GM-CSF were exposed to heat-inactivated *S. aureus *(10^7 ^cfu/well) or PGN (10 μg/ml) for 24 h, whereupon conditioned supernatants were collected and analyzed for TNF-α (A) and MIP-2 (B) expression by ELISA (mean ± SD). Microglial cell viability was assessed using a standard MTT assay and the raw OD_570 _absorbance values are reported (C). Results are representative of three independent experiments.

**Figure 4:**
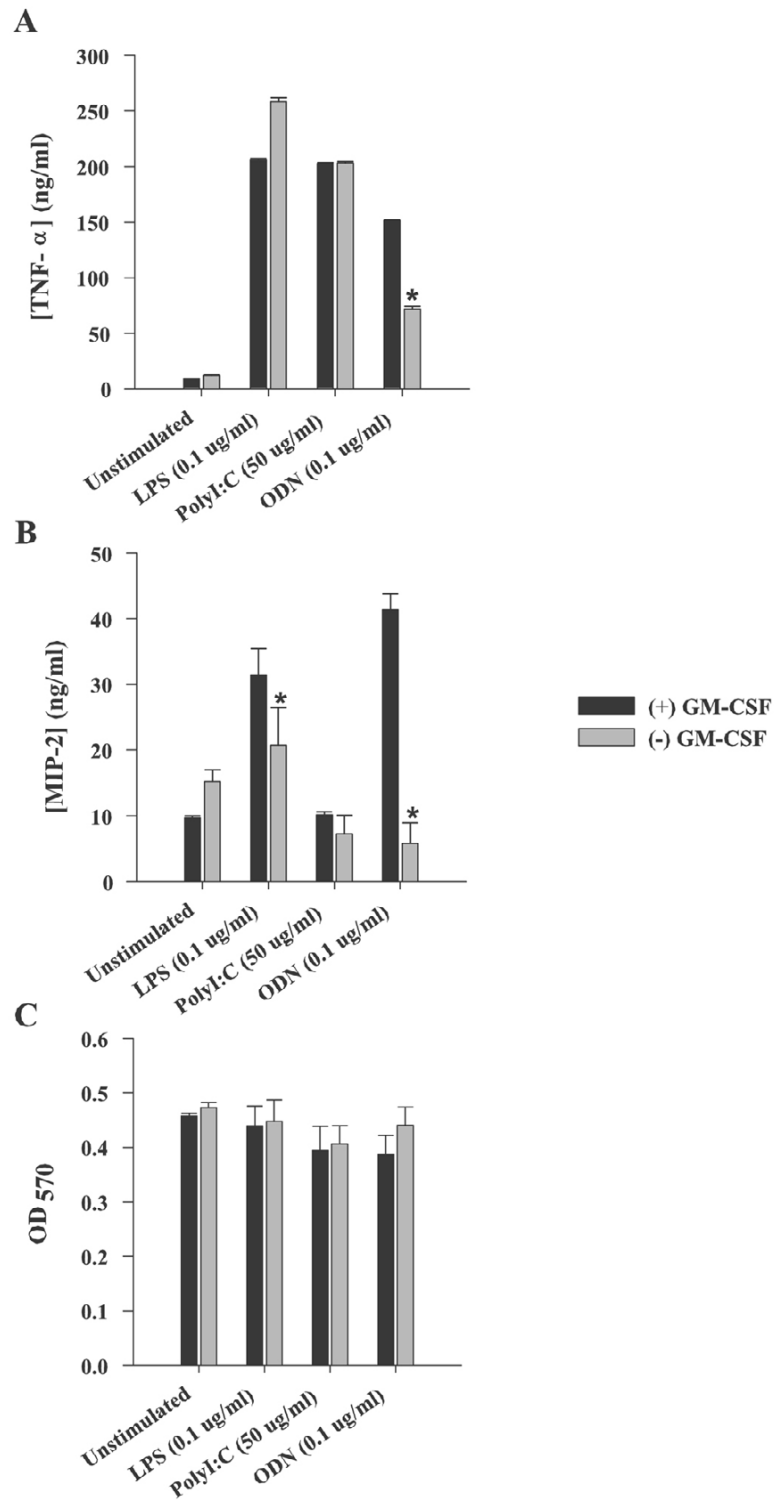
**Low dose GM-CSF influences microglial responsiveness to a downstream CpG-ODN (TLR9) stimulus**. Primary microglia expanded with (+) or without (-) GM-CSF were exposed to various concentrations of LPS, polyI:C or CpG-ODN for 24 h, whereupon conditioned supernatants were collected and analyzed for TNF-α (A) and MIP-2 (B) expression by ELISA (mean ± SD). Microglial cell viability was assessed using a standard MTT assay and the raw OD_570 _absorbance values are reported (C). Results are representative of three independent experiments. Asterisks denote significant differences between microglia propagated in the presence and absence of GM-CSF (* *p *< 0.05, ** *p *< 0.001).

It has been suggested that GM-CSF plays an important role in promoting the proinflammatory functions of primary microglia, since higher cytokine doses have been reported to induce the transcription of several proinflammatory mediators in neonatal microglia [14] as well as enhance the antigen presentation properties of adult microglia [10,14,15,32,40]. Although our previous studies using primary neonatal mouse microglia expanded in the presence of low dose GM-CSF did not detect significant constitutive proinflammatory mediator expression under resting conditions [11,20,23,41,42], we have not yet performed a detailed side-by-side comparison of activation profiles of microglia propagated in the presence/absence of GM-CSF during the mixed glial culture period. Therefore, in the present study, we evaluated proinflammatory mediator expression by microglia expanded with or without GM-CSF in response to a diverse array of PAMPs to establish the utility of low dose GM-CSF for microglial expansion without affecting downstream cellular responsiveness. The PAMPs evaluated included the gram-positive bacterium *S. aureus *and its cell wall component PGN, as well as polyI:C, LPS, and CpG-ODN. Similar to our previous results we found that propagating microglia in the presence of GM-CSF did not alter any constitutive proinflammatory mediator production or significantly affect the degree of responsiveness to either *S. aureus *or PGN (Figure 3). Specifically, TNF-α, MIP-2, and IL-12 p40 were produced to equivalent extents upon *S. aureus *and PGN activation by microglia expanded in the presence/absence of GM-CSF (Figure 3A and 3B and data not shown). To further examine the effects of low dose GM-CSF on microglial responses, we broadened our analysis to investigate several PAMPs acting through TLRs other than TLR2, in particular, polyI:C, LPS, and CpG-ODN, which stimulate microglia via TLR3, TLR4, and TLR9, respectively [12,43,44]. Of all of the PAMPs examined, responses to CpG-ODN were most affected by whether microglia had been expanded in the presence of low dose GM-CSF. Specifically, both TNF-α and MIP-2 production were significantly reduced in CpG-ODN treated cells that had no prior exposure to GM-CSF (Figure 4A and 4B). In contrast, responses to polyI:C and LPS remained unchanged or modestly affected, respectively (Figure 4), suggesting that low dose GM-CSF does not drastically alter microglial responsiveness to these PAMPs, in terms of the proinflammatory mediators examined here. Interestingly, polyI:C was not able to induce MIP-2 production in microglia above baseline levels regardless of GM-CSF exposure (Figure 4A, and 4B). Importantly, the concentrations of all stimuli used in this study did not adversely affect microglial viability, indicating that the exposure of microglia to GM-CSF during the expansion period did not sensitize cells to activation-dependent toxicity (Figures 3C and 4C).

### Microglial phagocytosis of bacteria is not affected by the presence of GM-CSF during the mixed glial culture period

**Figure 5:**
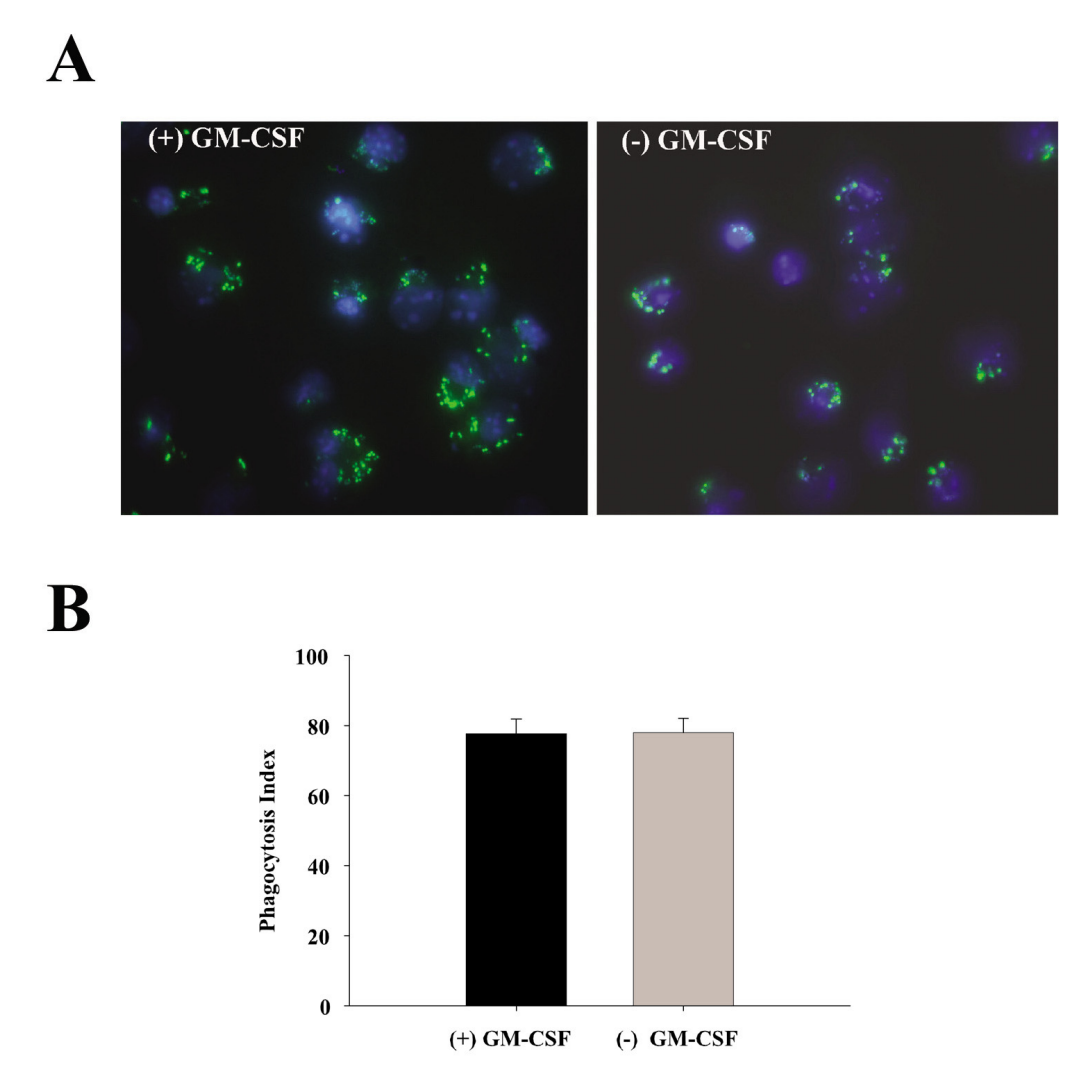
**Phagocytic activity of primary microglia is not affected by GM-CSF**. Primary microglia expanded either with (+) or without (-) GM-CSF were seeded onto 12 mm coverslips at 2 × 10^5 ^cells per coverslip and incubated overnight in 24-well plates. The following day, cells were treated with 2 × 10^6 ^heat-inactivated *S. aureus*-GFP (green) for 3 h and visualization of intracellular bacteria was detected using fluorescence microscopy (40×). Hoechst dye (blue) was used to visualize nuclei (A). In (B), the phagocytic index was calculated as the percentage of microglia which engulfed bacteria in a total of ten, 40 × microscopic fields. The results represent the mean ± SEM of two independent experiments.

Since microglia are the resident macrophages of the CNS parenchyma [45-47], phagocytosis of bacteria [11,48,49] or apoptotic cells [50] represent some of their primary functions. It has been shown that the phagocytosis rate of apoptotic cells was significantly higher in GM-CSF treated microglia as compared to unstimulated cells [16]. However, to our knowledge, no one has yet examined the consequences of GM-CSF on bacterial phagocytosis by microglia. In the present study we found that the expansion of primary microglia with GM-CSF did not affect their phagocytic activity. As shown in Figure 5A, both microglia cultured with or without GM-CSF were able to engulf GFP-labeled *S. aureus *to equivalent extents, where the phagocytic index (as determined by quantitating the number of microglia harboring intracellular bacteria) was not significantly different between the groups (Figure 5B). Although distinct phagocytic pathways are likely involved, our findings are similar to those of Fischer et al. (1993) where the percentage of microglia phagocytizing latex beads did not significantly differ following GM-CSF treatment [10]. Collectively, these results suggest that any effects of GM-CSF on microglial activation and bacterial phagocytosis are negligible at the low doses used as a culture medium supplement in our studies.

### Effects of low dose GM-CSF on the expression of microglial surface markers

Primary microglia can be differentiated from macrophages based on their characteristic staining pattern of CD11b^high ^and CD45^low ^[32,51-53]. Although it has been shown that GM-CSF used as either a culture medium supplement or a direct stimulus leads to a dramatic expansion in microglial numbers [10,13], an effect on CD11b expression was not demonstrated [13]. On the other hand, GM-CSF has been reported to inhibit the IFN-γ -induced expression of another surface marker, MHC class II, and as a consequence, modulate microglial APC functions [15,54]. However, Fischer at al. (1993) have shown that MHC class II expression is not altered on microglia grown in the presence of GM-CSF, whereas its expression is induced following IFN-γ treatment [10].

**Figure 6:**
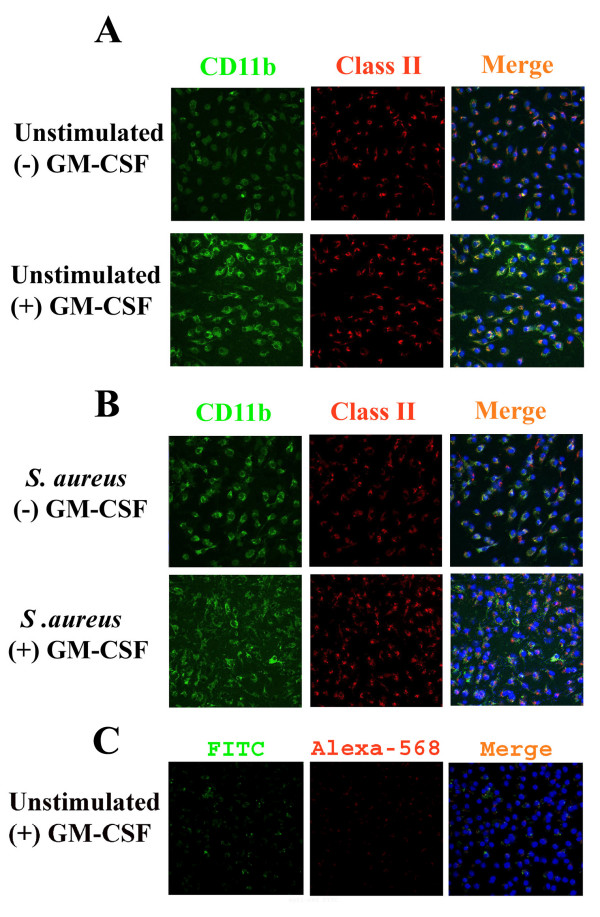
**Microglial CD11b, but not MHC class II expression, is influenced by low GM-CSF levels**. Primary microglia propagated either with (+) or without (-) GM-CSF were seeded onto 12 mm cover slips at 2 × 10^5 ^cells per cover slip and incubated overnight in 24-well plates. The following day, cells were either unstimulated (A) or treated with heat-inactivated *S. aureus *(10^7 ^cfu/well) (B) for 24 h, whereupon microglia were stained with CD11b-FITC (green) and MHC Class II-Alexa-568 (red). Nuclei were visualized with Hoechst dye (blue). Panel (C) depicts background staining with secondary antibodies only. Results are representative of two independent experiments.

**Figure 7:**
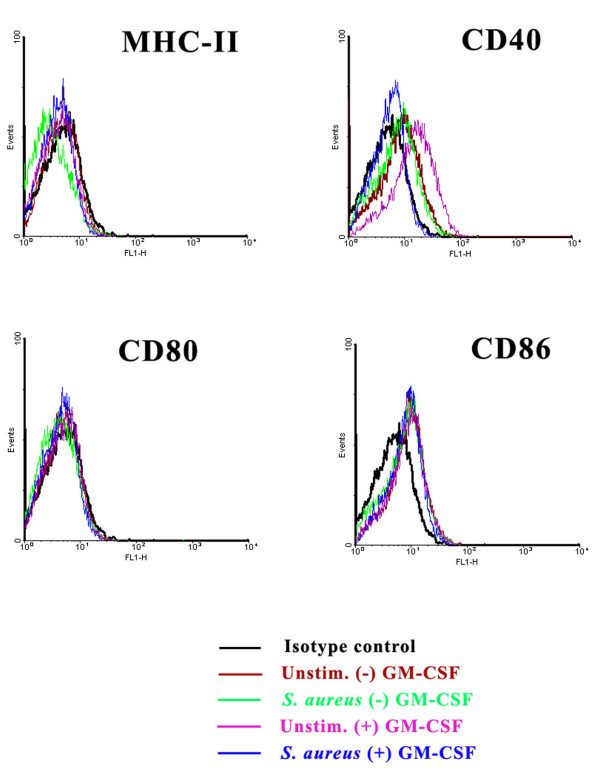
**Low dose of GM-CSF influences microglial CD40 expression in response to *S. aureus***. Primary microglia expanded either with (+) or without (-) GM-CSF were seeded into 6-well plates at 2 × 10^6 ^cells per well and incubated overnight. The following day, cells were either unstimulated or treated with heat-inactivated *S. aureus *(10^7 ^cfu/well) for 24 h, whereupon microglia were recovered and stained with MHC class II, CD40, CD80, or CD86 antibodies and subsequently with a FITC-conjugated secondary antibody for flow cytometric analysis. Microglia were stained with an isotype-matched control antibody to assess background staining. Results are representative of two independent experiments.

**Figure 8:**
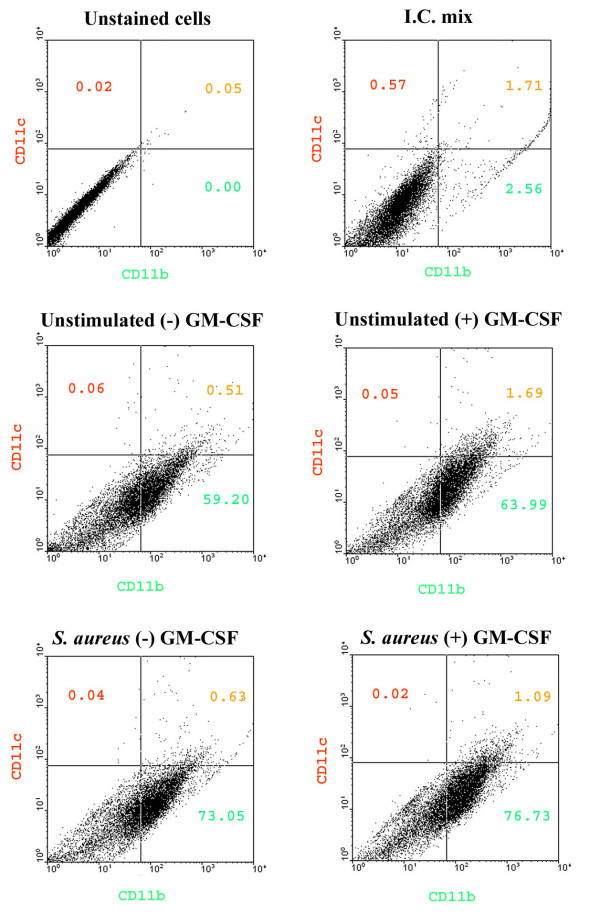
**CD11c expression is not induced in neonatal microglia propagated in low dose GM-CSF**. Primary microglia expanded either with (+) or without (-) GM-CSF were seeded into 6-well plates at 2 × 10^6 ^cells per well and incubated overnight. The following day, cells were either unstimulated or treated with heat-inactivated *S. aureus *(10^7 ^cfu/well) for 24 h, whereupon microglia were recovered and stained with CD11b-FITC and CD11c-PE-Cy7 for flow cytometric analysis. Cells were stained with isotype-matched control antibodies to demonstrate the extent of non-specific staining. The numbers shown in each quadrant represent the percentage of positive cells detected. Results are representative of two independent experiments.

In the present study we have demonstrated that constitutive CD11b expression was slightly enhanced in GM-CSF-expanded microglia compared to cells cultured without GM-CSF as determined by both immunofluorescence staining and FACS analysis (Figures 6A and 8, respectively). In addition CD11b expression was moderately increased following *S. aureus *stimulation regardless of whether microglia had been propagated in the presence or absence of GM-CSF (Figure 6A and 6B and Figure 8). With regard to MHC class II expression, considerable immunoreactivity was observed in "resting" microglia, similar to what has been reported by others with cultured neonatal microglia (Figure 6A) [10,14,54]. This constitutive MHC class II expression was not influenced by GM-CSF during the mixed glial culture period (Figure 6A). Unexpectedly, in contrast to what was observed with CD11b, *S. aureus *stimulation did not lead to a notable increase in MHC class II immunoreactivity and no obvious modulation by GM-CSF was observed (Figure 6B). This finding was independently confirmed by flow cytometric staining (Figure 7). The inability of *S. aureus *to augment MHC class II levels was unexpected, but could be explained by the fact that the constitutive MHC class II levels detected may not be subject to further increases following cell stimulation. Importantly, the degree of non-specific background staining observed was negligible (Figure 6C).

Since neonatal microglia exhibit a partially activated phenotype *in vitro*, as indicated by an intermediate expression level of co-stimulatory molecules in addition to MHC class II [52,55], we expanded our analysis to evaluate the expression of several co-stimulatory molecules including CD40, CD80 and CD86 by flow cytometry to determine the possible effects of low dose GM-CSF on microglial expression of these molecules. Similar to MHC class II, baseline CD80 and CD86 expression was not influenced by either GM-CSF or *S. aureus *(Figure 7). On the other hand, microglia propagated in the presence of low dose GM-CSF displayed elevated CD40 levels following *S. aureus *stimulation (Figure 7), whereas under resting conditions, CD40 levels were equivalent in microglia expanded with or without GM-CSF. Taken together, this data suggests that exposing microglia to low doses of GM-CSF during the mixed culture period does not lead to significant alterations in surface marker expression, with the exception of CD40, which may have an impact on microglial activation in the presence of CD40 ligands.

The adult brain parenchyma harbors a population of CD11b^+ ^myeloid precursors [51,56-58] that can be driven to differentiate into immature DCs by GM-CSF treatment as measured by the induction of cell surface markers such as DEC-205, CD11c, and CD80 [10,13,38,40]. When Fischer and co-workers (2001) incubated primary microglia from adult mouse brain with GM-CSF (50 ng/ml) for 5 days, approximately 30% of these cells expressed CD11c compared to < 0.5% of microglia in the initial population. In our culture system, microglia are exposed to GM-CSF for a period of longer than 10 days and this was not sufficient to induce CD11c expression since, when compared to isotype control staining, none of the CD11c signal detected could be attributed to specific binding in either the "resting state" or following *S. aureus *exposure (Figure 8). Collectively, these findings indicate that exposure of microglia to low dose GM-CSF during the mixed glial culture period does not induce the expression of the classical DC marker CD11c.

## Discussion

GM-CSF, which is a potent stimulator of microglia as well as macrophages and granulocytes, is usually detected in the brain following T cell infiltration [14,59,60] or produced by activation of astrocytes and/or endothelial cells [7,61]. The amount of GM-CSF released by these cells could be sufficient to induce a proinflammatory state of local microglia and/or their transformation into a DC- or macrophage-like cell. Although numerous studies have been performed examining the effects of GM-CSF on microglial morphology and function, often times these reports have produced conflicting results [10,14,16,54]. This likely stems from differences in GM-CSF treatment paradigms, species of microglial origin, and/or whether adult or neonatal cells are used. All of these issues coupled with the fact that the doses of GM-CSF applied to microglia have shown tremendous variability among individual studies, makes it quite difficult to compare results and arrive at general conclusions regarding the effects of GM-CSF on primary microglia. When we reviewed the literature we found that GM-CSF was mainly evaluated in two ways. In the first, GM-CSF was provided as a supplement during the initial mixed glial culture period [10,13,14,38,40], whereas in another group of studies, GM-CSF was added as a stimulus to purified microglia that had otherwise not been previously exposed to the growth factor [6,7,16,32,37,54]. Another variable is the dose of GM-CSF examined which has been tested over a very broad range, namely from 0.05–1 μg/ml or 1- 200 U/ml, which can be expected to have dramatic effects on the results obtained. In addition, the species from which primary microglia are procured and their maturational state, for example, mouse or rat newborn pups [6,13,14,16,40,54], adult animals [10,32,38], or even human fetal microglia [7] have been examined. Therefore, it is essential that the results obtained from these diverse experimental settings should be evaluated within the constraints of these parameters.

One main reason why scientists studying primary microglia have supplemented culture medium with GM-CSF is to procure sufficient numbers of microglia for downstream analysis, since GM-CSF has been shown to induce microglial proliferation [6,10]. Indeed, this modification has resulted in accelerated cell growth and increased cell yields, saving time and animal utilization. In our *in vitro *studies, we typically isolate primary mixed glial cells from newborn mice and culture them in medium supplemented with a relatively low dose of GM-CSF (0.5 ng/ml). Importantly, upon recovery of microglia from these cultures, cells are never exposed to exogenous GM-CSF again and are not utilized in experiments for at least 2 days following low dose GM-CSF withdrawal. With this modification, we were able to collect sufficient numbers of microglia for our experiments even after the third harvest, effectively reducing the need for additional microglia cultures. In addition, the main inflammatory responses of microglia following *S. aureus *stimulation, namely proinflammatory mediator production and phagocytosis, were not affected by propagating cells in the presence of GM-CSF (Figures 2 and 3). Interestingly, exposure of microglia to GM-CSF modestly influenced proinflammatory mediator production following LPS stimulation, whereas responses to polyI:C were not affected (Figure 4). It has been well established that both of these PAMPs activate microglia via TLR4 and TLR3, respectively, leading to GM-CSF production [62-66], as well as the priming ability of GM-CSF to augment LPS-induced TNF-α production in monocytes [67] and alveolar macrophages [68]. However, to the best of our knowledge, the current study represents the first report investigating the effects of low dose GM-CSF on microglial responses to TLR4 and TLR3 ligands. Importantly, based on the finding that low dose GM-CSF does not dramatically alter microglial proinflammatory mediator expression in response to a diverse array of PAMPs, we propose that our cell culture procedure may be applicable to a wide range of studies investigating the immune properties of primary mouse microglia without concerns of drastically transforming cellular responsiveness. However, a word of caution must be mentioned, in that not all PAMP recognition is resilient to low dose GM-CSF, as demonstrated by our CpG-ODN findings discussed below.

Several researchers have reported that microglia express TLR9 and respond to the TLR9 agonist CpG-ODN with the robust production of numerous proinflammatory mediators as well as increased expression of immune receptors [12,69-71]. Moreover, recent evidence demonstrating that CpG-ODN stimulated microglia induce neuron cell death in a co-culture paradigm has suggested a link between TLRs and neurotoxicity and neurodegeneration [69]. On the other hand, we have reported that alternative TLR agonists such as *S. aureus*, PGN and LPS lead to a time-dependent decrease in TLR9 mRNA expression [20], which might be explained as a compensatory mechanism for microglia to downregulate cellular activation pathways. A recent study has shown that GM-CSF (20 ng/ml) in combination with either CpG- or non-CpG-ODN was capable of inducing several chemokines in primary human monocytes [72], as well as in lymphoma and neuroblastoma models [73,74]. Although the methods used in these studies are quite distinct compared to our experimental design, they still support our findings that GM-CSF exacerbates microglial activation following CpG-ODN stimulation (Figure 4). Our results highlight the importance of experimental culture conditions and the potential effects of GM-CSF, even at very low doses, should be considered while investigating the consequences of GpG-ODN on microglial activation. The mechanism(s) responsible for this observed synergistic effect between GM-CSF and CpG-ODN remain to be clarified with further studies. Importantly, in this study microglia expanded in the presence/absence of GM-CSF expressed equivalent levels of TLR9 mRNA under resting conditions (data not shown), suggesting that the impaired responsiveness of GM-CSF (-) microglia to subsequent ODN stimulation is likely not the result of reduced TLR9 expression.

One of the main concerns reported with GM-CSF treatment of primary microglia cultures is its ability to induce microglial differentiation towards a DC phenotype [10,15,40]. GM-CSF can be produced by glial cells following inflammatory or infectious diseases of the brain [75-78]; therefore, it was postulated that GM-CSF may modulate the generation and maturation of DCs from microglia in the context of CNS disease [38]. Although our experiments demonstrate that low dose GM-CSF does not lead to overt alterations in the functional properties of microglia to diverse bacterial stimuli, cytokine treatment does induce morphological changes characteristic of DC-like cells. Specifically, we found that unstimulated microglia expanded in the presence of GM-CSF displayed an increased number of dendritic processes and were enlarged and more adherent compared to cells that had not been exposed to exogenous growth factor. Despite these phenotypic differences, we were not able to uncover any functional implications for these changes and no CD11c induction on GM-CSF exposed microglia could be demonstrated. Therefore, additional studies are warranted to further investigate the consequences, if any, of this morphological transformation of microglia following low dose GM-CSF exposure. A general consensus that can be inferred from the literature, and is in partial agreement with the results presented here, is that GM-CSF can lead to the transition of microglia into a DC-like phenotype, although the magnitude of these changes appears to be influenced by the dose of GM-CSF cells are exposed to.

The expression of other surface molecules including MHC class II, CD80, and CD86 were not affected by exposure of microglia to low dose GM-CSF. The expression of these molecules has been shown by others to be elevated in neonatal microglia, where cells exhibit a partially activated phenotype in culture [15,52,55]. In *ex vivo *isolated adult microglia, GM-CSF stimulation did not alter the expression of MHC class II, CD40, or CD86 but did induce morphological changes [15,54]. In contrast to our previous report demonstrating that *S. aureus *stimulation induced MHC class II and co-stimulatory molecule expression in the N9 microglial cell line [11], we found in the present study that the levels of MHC class II, CD80, and CD86 were not augmented in primary microglia following *S. aureus *stimulation. However, CD40 expression was dramatically upregulated following *S. aureus *stimulation in microglia that were expanded in the presence of GM-CSF. We propose that one explanation for these contradictory findings may lie in the nature of the cell types examined. For example, primary neonatal microglia in culture may inherently express higher constitutive levels of these molecules such that further activation will not result in an obvious increase in their expression. Alternatively, the length of time required to detect activation-dependent increases in surface marker expression in primary microglia may be longer than that observed for the N9 cell line; however, this possibility seems less likely. Another potential explanation is that the concentration of *S. aureus *required to increase MHC class II and co-stimulatory molecule expression may differ between primary microglia and the N9 cell line. Since we only examined one dose of *S. aureus *in the present study it remains possible that titration of higher bacterial concentrations may have elicited an increase in surface marker expression, although this possibility remains speculative. The finding that MHC class II levels did not increase in *S. aureus *stimulated microglia when assayed using two independent approaches (i.e. immunofluorescence and flow cytometry staining) provides additional support to establish the validity of our findings.

Another important factor to consider is the nature of how primary microglia are propagated during *in vitro *culture. Specifically, in our studies, microglia and astrocytes are maintained as mixed cultures during the expansion period. Therefore, it is important to acknowledge that the coordinated effects of endogenous astrocyte-derived factors in combination with exogenous GM-CSF may influence the properties of recovered microglia. As an extension, it is not surprising that different microglial phenotypes are observed when cells are directly purified from the brain parenchyma and cultured in isolation. In addition, compounded with the addition of high levels of exogenous GM-CSF used in other studies, it is not unexpected that microglia assume different biological and functional properties. We propose that the maintenance of primary microglia initially as mixed cultures with astrocytes is more reminiscent of the natural milieu these cells are exposed to *in vivo *and represents an acceptable model to study the immunological properties of microglia, although we clearly recognize the potential for any *in vitro *findings to not directly equate to *in vivo *responses due to the artificial nature of the former. However, the examination of microglia in isolation provides a more simplified approach to investigate the effector functions of these CNS phagocytes in the absence of confounding effects by surrounding cell types.

## Conclusion

Our findings demonstrate that supplementation of neonatal mouse mixed glial cultures with low dose GM-CSF successfully maintained microglial expansion and did not lead to overt alterations in the functional responses of microglia following stimulation with several PAMPs of importance during CNS bacterial and viral infections including PGN, LPS, polyI:C, as well as *S. aureus*. This suggests that our culture paradigm may be successfully used as a method to procure larger quantities of microglia without significantly affecting their downstream responses to microbial stimuli. However, it is important to note that differences in CpG-ODN responsiveness as well as the transition towards a DC-like phenotype were observed in microglia propagated in the presence of low dose GM-CSF, the functional implications of which are currently unknown.

## Abbreviations

APC (antigen presenting cell); CD (cluster of Differentiation); CFU (colony-forming units); CNS (central nervous system); DC (dendritic cell); DMEM (Dulbecco's modified eagle medium); ELISA (enzyme linked immunosorbent assay); FACS (fluorescence activated cell sorting); FBS (fetal bovine serum); FITC (fluorescein isothiocyanate); GFP (green fluorescence protein); IFN-γ (interferon gamma); IL-12 p40 (interleukin-12 p40); GM-CSF (granulocyte-macrophage colony-stimulating factor); LPS (lipopolysaccharide); M-CSF (macrophage colony-stimulating factor); MHC (major histocompatibility complex); MIP-2/CXCL2 (macrophage inflammatory protein-2); MTT (3- [4,5-dimethylthiazol-2-yl]-2,5-diphenyl-tetrazolium bromide); ODN (oligonucleotide); OPI (oxalacetic acid, pyruvate, insulin); PAMP (pathogen associated molecular pattern); PGN (peptidoglycan); PBS (phosphate buffered saline); polyI:C (polyinosine-polycytidylic acid);*S. aureus *(*Staphylococcus aureus*); TLR (Toll-like receptor); TNF-α(tumor necrosis factor-alpha).

## Competing interests

The author(s) declare that they have no competing interests.

## Authors' contributions

Both NE and TK directed the work and prepared the manuscript.
